# Hybrid method for estimating lung ventilation from CT by combining intensity and motion information

**DOI:** 10.1002/mp.17787

**Published:** 2025-03-30

**Authors:** Paris Tzitzimpasis, Mario Ries, Bas W. Raaymakers, Cornel Zachiu

**Affiliations:** ^1^ Department of Radiotherapy University Medical Center Utrecht Utrecht The Netherlands; ^2^ Imaging Division UMC Utrecht Utrecht The Netherlands

## Abstract

**Background:**

Functional lung imaging modalities allow for capturing regional lung ventilation information. Computed Tomography based ventilation imaging (CTVI) has been proposed as a surrogate modality that relies on time‐resolved anatomical data and image processing. However, generating accurate ventilation maps using solely computed tomography (CT) image information remains a challenging task, due to the need to derive functional information of ventilation from anatomical observations.

**Purpose:**

We introduce the hybrid estimation of computed tomography obtained respiratory function (HECTOR) method that consists of two components: a volume‐ and a density‐based ventilation estimate. For the first component, a deformable image registration (DIR)—based solution for accurate volumetric CTVI generation is proposed, integrating the physical characteristics of the lung deformations in its design. For the second component, an already established air‐tissue density model is used. Furthermore, a novel method is developed for combining the two components.

**Methods:**

The proposed method consists of four principal steps: (1) Application of a specially tailored DIR algorithm to estimate respiratory motion between inhale and exhale phases. (2) Conversion of the motion information to volumetric change maps using a variation of the Jacobian determinant method. (3) Computation of a HU‐based method that estimates the local product of air‐tissue densities. (4) Combination of the metrics estimated in steps 2 and 3 by means of a smooth minimum function.

The proposed approach is validated using the publicly available VAMPIRE dataset consisting of two subgroups: 25 subjects scanned with Galligas 4DPET/CT and 21 subjects scanned with DTPA‐SPECT. Another dataset of 18 patients available at The Cancer Imaging Archive (TCIA) was used for further validation. All datasets contain inhale/exhale CT scans paired with ground‐truth ventilation images (RefVIs). The CTVIs generated by the proposed HECTOR method were tested against the RefVIs using the Spearman correlation coefficient and Dice overlap of low‐ and high‐function lung (DSC‐low and DSC‐high, respectively).

**Results:**

The proposed method achieved mean Spearman, DSC‐high and DSC‐low coefficients of 0.62, 0.55, and 0.59 on the Galligas PET subgroup and 0.49,0,48, and 0.50 on the DTPA‐SPECT subgroup of the VAMPIRE dataset. This performance was better than the highest performing method reported in the original challenge. The same metrics for the TCIA dataset were 0.66, 0.60, and 0.60. The proposed hybrid ventilation method achieved higher Spearman correlation scores than the individual volume‐ and density‐based components in all datasets. Additionally, the use of the specially tailored DIR algorithm was found to achieve higher scores than previously reported volume‐based methods.

**Conclusions:**

Our work provides a novel processing workflow for CT ventilation imaging that can consistently generate ventilation maps with high fidelity compared to reference approaches. This study also provides further insights into the benefits of combining different types of information to model the complex dynamics of respiratory function. Such information can be useful for potential applications in radiation therapy treatment planning and thoracic dose–response assessment.

## 1 INTRODUCTION

Lung cancer is the leading cancer type in terms of mortality rates worldwide, accounting for 18% of cancer‐related deaths globally^1^ and 21% in the United States.^2^ More than 50% of lung cancer patients undergo radiotherapy as part of their treatment.^3^ At the same time, a significant percentage of those (5%–25%) experience radiation induced lung injury^4^ which therefore limits the maximum delivered dose. Conventional radiotherapy treatment plans assume homogeneous function throughout the lungs being treated as organs at risk. However, both in healthy individuals and especially in patients with lung disease, pulmonary function can present a high degree of spatial heterogeneity.^5^ Functional lung avoidance in radiation therapy (FLART) attempts to selectively minimize the delivered dose to highly functional lung tissue without compromising target coverage and organ at risk constraints. A number of clinical trials have established the clinical feasibility of FLART^6^, ^7^, ^8^ while the potential reduction of grade ≥2 pneumonitis has also been demonstrated.^9^ Along similar lines, it has been reported that measures of lung function can be effectively used in combination with dose metrics as predictors of lung toxicity after radiation therapy^10^, ^11^ suggesting dose‐function constraints could be more relevant than their dose‐volume counterparts in treatment planning. Previous results^12^ have shown reduced grade ≥2 radiation pneumonitis rates compared to historical control. The results of a recent randomized clinical trial^13^ have also indicated reduced toxicity and less pulmonary function test (PFT) decline for non‐small cell lung cancer (NSCLC) patients treated with conventionally fractionated functional avoidance compared to the standard of care.

However, these planning strategies rely on a spatially resolved functional assessment of the lung. Imaging modalities that can provide measurements of the local pulmonary function include single photon emission computed tomography^14^ (SPECT) with technetium‐99m (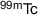), positron emission tomography^15^ (PET) with gallium‐68 (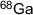), and magnetic resonance imaging^16^ (MRI) with hyperpolarized helium‐3 (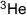). These approaches are based on the observation of labeled tracer gas. On the other end of the spectrum, CT‐ventilation imaging (CTVI) is an image processing modality that utilizes one or more CT images and employs a series of postprocessing steps in order to provide surrogate lung function maps. Although CTVI is a qualitative and indirect assessment of the functional state of the lung, its synthetic nature allows for circumventing the need for specialized equipment, the use of exogenous contrast agent and the additional costs associated with the aforementioned nuclear medicine methods while providing functional images of the same resolution as the original CT scans. Furthermore, given that four‐dimensional CT (4DCT) scans are part of most radiotherapy planning and/or treatment workflows, CT‐ventilation significantly alleviates the burden of undergoing additional scanning sessions. At the same time, the functional image is by construction aligned with the CT image from which it is generated without requiring the additional step of, possibly multimodal, deformable alignment which can be error‐prone.

In the past, a variety of methods have been proposed for accurately retrieving functional information from CT scans. In one of the first approaches,^17^ lung voxels were modeled as compartments consisting of a mixture of watery tissue and air to derive a linear relationship between Hounsfield units (HU) in a CT and fractional air content. The change in fractional air content between inspiration and expiration can then be measured as a surrogate for ventilation. This paradigm uses deformable image registration (DIR) to track HU changes^18^ of corresponding voxels between extreme respiratory states. Such models are often referred to as DIR‐HU methods. A different approach^19^ classifies tissue functionality based on the local volumetric changes estimated between two respiratory phases. Those so‐called volume‐based methods usually rely on the computation of the deformation estimated between the full inhale/exhale phases. Subsequently, the Jacobian determinant of the deformation is computed, which quantifies the amount of local tissue expansion. Similarly, the N‐phase local expansion ratio (LER‐N) method^20^ has been proposed for utilizing intermediate respiratory phases in order to account for out‐of‐phase ventilation patterns. Both HU‐based and Jacobian‐based methods have been used and demonstrated different levels of correlation with nuclear medicine methods, such as SPECT,^18^ Galligas PET,^21^ hyperpolarized 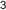 He, and 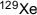 MRI.^22^ Previous studies have further investigated non‐DIR based approaches using solely HU values.^23^ Recently, deep learning methods have also been employed for the synthesis of ventilation and perfusion maps,^24^, ^25^ reporting good agreement with DIR‐based methods^26^ and significant voxel‐wise correlations with 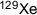 MRI.^27^ While this direction of research seems promising, the main obstacle in the adoption of deep learning methods in clinical practice is their moderate adaptability to different individuals. Given the heterogeneity of pulmonary function distribution across patient populations with underlying respiratory conditions, selecting representative training datasets can be challenging.

Here, we introduce the HECTOR, a hybrid method that employs both volume and HU‐based estimates to assess regional ventilation capacity. The hypothesis is hereby that volume and HU based methods display complementary strengths and weaknesses and that by combining them, the robustness for functional pulmonary assessment can be improved. Our contribution is twofold:

1. We introduce a novel image registration algorithm that is specifically tailored for robust volumetric change estimation. In the proposed registration method, explicit constraints are introduced that control the magnitude and spatial smoothness of the estimated volume changes.
2. We introduce a general framework for merging information from two different ventilation metrics. We use this framework to combine the volume‐based method with a purely HU‐based approach.

## 2 METHODS

### 2.1 Image datasets

We used data available from the Ventilation And Medical Pulmonary Image Registration Evaluation (VAMPIRE) challenge,^28^ a public multi‐institutional dataset encompassing 50 4DCT datasets paired with corresponding reference ventilation images (RefVI's). The full challenge consists of three separate studies. Study 1^29^ included 25 lung cancer patients imaged with Galligas 4DPET/CT before radiation therapy. The 4DCT resolution was $1.07\times 1.07\times 5{\mathrm{mm}}^{3}$. The in‐plane resolution of the PET scans was $2.86\times 2.86{\mathrm{mm}}^{2}$ with a slice thickness of 3.3 mm. In order to reduce noise, time‐averaged (3D) scans were derived that were co‐registered to the time‐averaged anatomy. Study 2^19^ includes 4DCT and Xenon CT images co‐registered to the exhale phase of the 4DCT. This study was performed on 4 healthy sheep and was excluded from our investigation since our aim is the modeling of human ventilation. Study 3^30^ includes 21 lung cancer patients with their treatment planning 4DCT (in‐plane resolution of $0.97\times 0.97{\mathrm{mm}}^{2}$ and slice thickness of 2‐3 mm). Diethylenetriamine pentaacetate acid (DTPA)‐SPECT scans were obtained on a different day (4± 5 days later) with isotropic 8 mm resolution. The DTPA‐SPECT scans were then linearly re‐sampled to the 4DCT dimensions and rigidly registered to the time‐averaged anatomy. For all the datasets used, lung segmentations of the inhale and exhale respiratory phases were obtained using ITK‐snap.^31^

In order to further evaluate the proposed CTVI algorithm, we also employed data from a study^32^ that is publicly available on TCIA. Part of the data was originally used^33^ for the purpose of comparing different CTVI methods and assessing the effect of imaging on the accuracy of the resulting functional images. During the study, 20 lung cancer patients underwent breath‐hold CT (BHCT), free‐breathing 4DCT and Galligas PET ventilation scans. Patients 2 and 3 were discarded due to minimal motion between the inhale/exhale phases, a limitation that is also reported in the original dataset description. For patient 5, the inhale and exhale scans have a different number of slices and had to be manually resized. A manual rigid registration step was applied to align the exhale BHCT with the functional scan after defining lung segmentation masks on both of them (using ITK‐snap). Similar to the original study,^33^ the PET ventilation scans were also post‐processed with a median filter of size 7 to reduce noise levels.

### 2.2 Image registration

Image registration provides a voxel‐wise transformation that aligns one image to another. An image registration algorithm consists of three main parts: the data‐similarity metric, the transformation model, and the optimization scheme. The similarity metric is a measure that quantifies the similarity between the given images. The transformation model aims to restrict the space of solutions by enforcing physical properties that the deformation has to abide by. Our registration model relies on the generalized div‐curl (GDC) algorithm.^34^ Registration is formulated as a minimization problem of the following variational

(1) minui∫ΩD(F,M,u⃗)+α1divu⃗2+α2∇divu⃗2+β∇curlu⃗2dx⃗∫Ω

here $D(F,M,\vec{u})$ is the data‐similarity metric which for our work was chosen to be the Local cross‐correlation^35^ (LCC). The three regularization terms appearing are:

1. A divergence magnitude penalty with weight $\alpha_{1}$ that penalizes the volumetric change magnitude. This term introduces some degree of selectivity, limiting redundant expansions, or contractions.
2. A divergence gradient penalty with weight $\alpha_{2}$ that penalizes spatial inhomogeneities of the volume expansions. This is a biologically motivated choice since we expect breathing expansion/contraction patterns to exhibit some degree of spatial homogeneity.
3. A curl regularization with weight β. This term is introduced on the basis of computational stability. It is used in order to provide regular motion fields.

As in the original implementation, the primary variables being estimated are not the traditionally used Cartesian components of the deformation field but the divergence and curl components which from now on we denote as $f^{1}=\text{div}\vec{u}$ and $f^{i}\left(\vec{x}\right)={\left(\text{curl}\;\vec{u}\right)}^{i-1}$ with i=2,3,4. Before registration, the following preprocessing steps were applied:

1. Cropping/padding such that the minimum distance between a lung voxel in the inspiration phase and the image boundary is 10 voxels. Padding was needed, for example, when the lower part of the lung was truncated.
2. Linear resampling to a uniform grid of size 128×128×128.
3. Intensity normalization between 0 and 1.

The above steps are essential for two reasons. First, it is important to standardize image dimensions and intensities to ensure that the set of chosen hyperparameters (regularization weights, number of resolution levels, and so forth.) is optimal for every input. Standardizing the inputs with respect to the anatomy of interest is therefore highly advantageous. Second, the specific size of 128 voxels was chosen on the basis of being sufficient to capture the regional variations in lung function. Considering that the linear dimensions of a pair of lungs are approximately 20 cm in length, width, and depth^36^ and the lungs in our cropped/resampled images can fit in a 100×100×100 voxel cube, the final voxel size in each dimension is of the order of 2 mm. It has been argued^37^ that the smallest functionally meaningful volume corresponds to the size of the gas exchange unit, the acinus, which is of the order of 180 ${\mathrm{mm}}^{3}$. The same study argues that a resolution of $10\times 10\times 10{\mathrm{mm}}^{3}$ is likely an appropriate scale for imaging of physiological processes related to gas exchange. With our choice of image dimensions, we are therefore well within those limits. At the same time, downsampling decreases image noise, which can be a significant source of error.

In our experience, many image registration algorithms are prone to erroneously estimating large local expansions/contractions, resulting in physiologically implausible ventilation estimations. For this purpose, we also included an additional constraint, limiting the allowed estimated divergence to $f^{1}\in \left[-0.5,0.5\right]$. This enforces that the local tissue contraction ratio $|\Delta V/V_{0}|$ is less than 2. We have empirically observed that contractions larger than this threshold are rare even in cases with large initial displacements and often occur due to numerical errors or image artifacts that cause irregular motion patterns. Such constraints have also been employed by previous methods^38^, ^39^ in order to avoid the estimation of physiologically implausible deformations. Finally, the algorithm parameters appearing in Equation (1) were tuned using the training data of the VAMPIRE dataset and were set to $\alpha_{1}=0.1$, $\alpha_{2}=2$, β=0.05 while a weighting kernel of 1.5 voxels was used for the calculation of the LCC similarity metric. More information on the effect of these parameters can be found in the original publication.^34^

### 2.3 Ventilation metrics

Deriving local lung ventilation estimates requires the use of a ventilation metric that translates image and/or deformation information into functional scalar maps. In the present work, we introduce a combination of two existing volume‐based^18^ and HU‐based^23^ ventilation metrics. We begin with a description of the former, volume‐based method. Consider a lung voxel of the exhale image with coordinates ${\vec{x}}_{\text{exhale}}$ and suppose that the spatial transformation $T(\vec{x})$ is known such that

$${\vec{x}}_{\text{exhale}}={\vec{x}}_{\text{inhale}}+T\left({\vec{x}}_{\text{inhale}}\right).$$

The specific volume change computed on the inhale anatomy is given by

(2) $$\begin{array}{c} {\text{sVol}}^{*}\left({\vec{x}}_{\text{inhale}}\right)=1-\text{Jac}\left({\vec{x}}_{\text{inhale}}\right)≃-\text{div}\vec{u}\left({\vec{x}}_{\text{inhale}}\right)=-f^{1}, \end{array}$$

where we used the first‐order approximation of the Jacobian determinant $\text{Jac}\left(\vec{x}\right)≃1+\text{div}\vec{u}\left({\vec{x}}_{\text{inhale}}\right)$. In this way, we observe that one of the registration parameters, the divergence $f^{1}$, admits a physical interpretation that is pertinent to local lung ventilation. The sensitivity of the Jacobian determinant to small perturbations of the deformation field is a known shortcoming of Jacobian‐based computations that has been discussed in previous studies.^39^ Truncating higher order modes makes our estimation less prone to numerical errors, while maintaining sufficient degrees of freedom to capture the spatial inhomogeneity of local ventilation. What is more, using the registration parameter $f^{1}$ in the ventilation metric computation, we avoid the need for derivative based postprocessing calculations, thus restricting error propagation. The next step is transporting the specific volume change to the exhale image by applying a forward transformation to the specific volume change approximation of Equation (2) thus giving us the simple expression for the volume‐based part of the ventilation metric:

(3) $$\begin{array}{c} {\text{CTVI}}_{\text{vol}}=-f^{1}\left({\vec{x}}_{\text{inhale}}+\vec{u}\left({\vec{x}}_{\text{inhale}}\right)\right) \end{array}$$

The HU‐based component of our ventilation metric follows an established method^23^, ^33^ for deriving measures of local blood‐gas exchange based purely on HU values:

(4) $$\begin{array}{c} {\text{CTVI}}_{\text{HU}}=\frac{{\text{HU}}_{\text{ex}}\left(\vec{x}\right)}{-1000}\times \frac{{\text{HU}}_{\text{ex}}\left(\vec{x}\right)+1000}{1000} \end{array}$$

where ${\text{HU}}_{\text{ex}}$ is the HU value at voxel $\vec{x}$ of the exhale scan. Overall, ${\text{CTVI}}_{\text{HU}}$ represents the product of local tissue and air fractional densities which are given by $\rho^{\text{air}}=-{\text{HU}}_{m}\left(\vec{x}\right)/1000$ and $\rho^{\text{tissue}}=1-\rho^{\text{air}}=\left({\text{HU}}_{m}\left(\vec{x}\right)+1000\right)/1000$. The model predicts a maximum ventilation capacity when $\rho^{\text{air}}≃\rho^{\text{tissue}}≃0.5$ and minimum when either $\rho^{\text{air}}≃0$ or $\rho^{\text{tissue}}≃0$. In this way, ${\text{CTVI}}_{\text{HU}}$ serves as a simple model for physiological ventilation which is the result of blood‐gas exchange and therefore requires comparable proportions of both blood (tissue) and air. In order to improve noise robustness, a median filter was applied with a patch size of five voxels as a postprocessing step.

### 2.4 Hybrid ventilation metric

We now describe our strategy for fusing two ventilation metrics in a consistent way that is independent of physical units and rescalings used in the derivation of each independent component. This property, that we refer to as *local rescaling invariance* ensures that given two ventilation maps A and B, their combined ventilation F(A,B) satisfies

(5) $$\begin{array}{c} F(A,B)=F(f(A),g(B)) \end{array}$$

for arbitrary monotonically increasing scalar functions f,g. This way, it is only the global ordering of voxels in A and B that affects F(A,B) and not the corresponding voxel values themselves. In order to achieve that, we first convert the given ventilation maps A and B to the corresponding *ordering maps* O(A) and O(B). The value $O\left(A\right)\left(\vec{x}\right)$ at voxel $\vec{x}$ is a positive integer denoting the order of the element $A(\vec{x})$ within the ventilation map. Therefore, the value 1 is ascribed to the voxel with the smallest ventilation value and the value N to the voxel with the highest ventilation where N is the number of voxels in the lungs. We note that O(A) contains the same functional information as A. For consistency, we proceed by linearly rescaling the ordered data between 0 and 1. We denote those normalized maps as $O_{N}\left(A\right)$. Unless this final step is performed, the maximum value in O(A) depends on the number of lung voxels which is in general different for every image.

Despite having ensured the local rescaling invariance property, there still exist multiple ways in which the two ventilation metrics can be combined. To motivate our modeling choice, we begin by noting that pulmonary function is expected to be homogeneous in healthy individuals.^40^ Underlying lung conditions, such as fibrosis or emphysema, can compromise regional ventilation capacity or even diminish it entirely. Such defects might appear to have imbalanced air/blood ratio (low ${\text{CTVI}}_{\text{HU}}$), exhibit small respiration induced volume change (low ${\text{CTVI}}_{\text{vol}}$) or both. Therefore, it is desirable to obtain a combined metric that highlights dysfunctional regions identified by either ${\text{CTVI}}_{\text{HU}}$ or ${\text{CTVI}}_{\text{vol}}$. An efficient way to achieve this, is by computing the voxelwise minimum between the two given ventilation metrics. In order to avoid discontinuities in the final ventilation maps, we chose to use the smooth minimum function. Therefore our final hybrid HECTOR metric is given by

(6) $$\begin{array}{c} {\text{CTVI}}_{\text{HECTOR}}=S_{\min}\left(O_{N}\left({\text{CTVI}}_{\text{HU}}\right),O_{N}\left({\text{CTVI}}_{\text{vol}}\right)\right)\\ \text{where}\;\;\;\;\;S_{\min}\left(A,B\right)=\frac{A\cdot e^{-A}+B\cdot e^{-B}}{e^{-A}+e^{-B}} \end{array}$$

**FIGURE 1:**
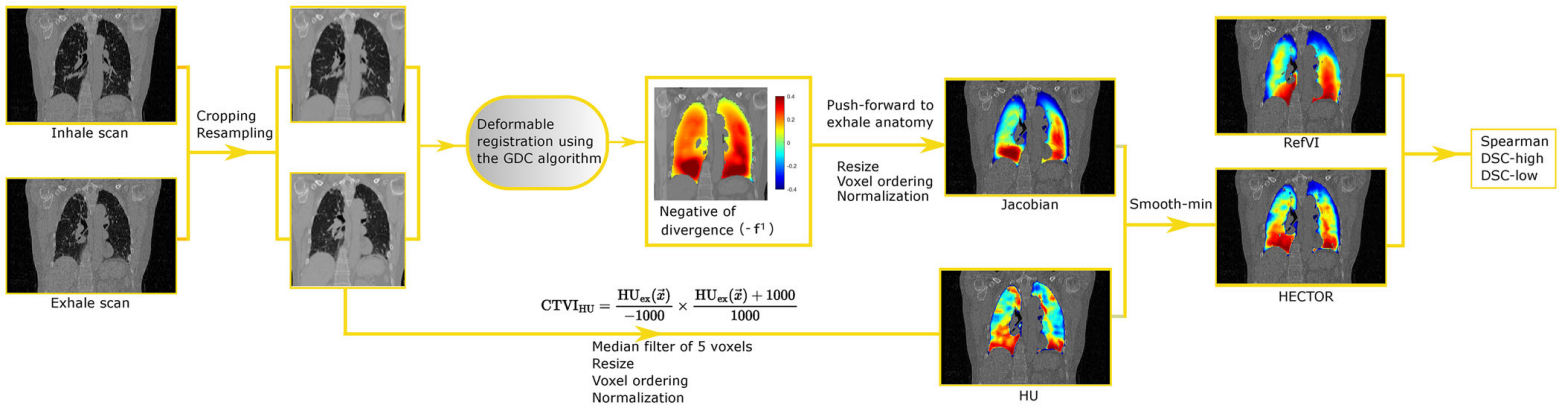
Illustration of the proposed workflow used to generate and validate the fused ventilation map ${\text{CTVI}}_{\text{HECTOR}}$. The normalization operation refers to linear rescaling of the data in the interval [0,1]. We observe that the smooth minimum function at the last step highlights areas of low ventilation estimated from either one of the two components (volume and HU‐based). CTVI, CT‐ventilation imaging; HU, Hounsfield unit.

Overall, the proposed workflow is summarized in Figure 1. We remark that, although the estimation of the individual HU and Jacobian components is carried out in the cropped/resampled images, the final output is resampled to the original exhale anatomy grid.

### 2.5 Evaluation

In accordance with the original VAMPIRE challenge evaluation pipeline, we used the voxel‐wise Spearman correlation coefficient $r_{s}$ and Dice overlap of the 25% highest and lowest functional regions (${\text{DSC}}_{\text{high}}$ and ${\text{DSC}}_{\text{low}}$). Similar to the data grouping of the original challenge, we divided the datasets into a training group (15 subjects) and a validation group (31 subjects). The first group was used for calibrating algorithm parameters such as regularization orders/weights, the number of resolution levels, image dimensions, and ventilation metric parameters. The second group was used for evaluation. The same metrics were used for the evaluation of the tested algorithms on the TCIA dataset. The parameters used for all three tested methods were kept the same throughout all experiments.

## 3 RESULTS

### 3.1 VAMPIRE dataset

**FIGURE 2:**
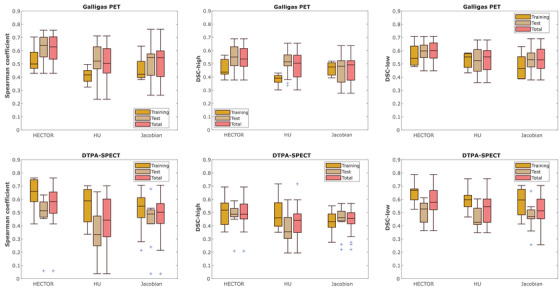
Results from the VAMPIRE challenge data. The top row shows the results on the PET substudy and the bottom row those of the DTPA‐SPECT. We observe that the median Spearman coefficient using the HECTOR metric is consistently higher than both individual components (Jacobian and HU) for all data groups. HECTOR, hybrid estimation of computed tomography obtained respiratory function; HU, Hounsfield unit; PET, pulmonary function test.

**FIGURE 3:**
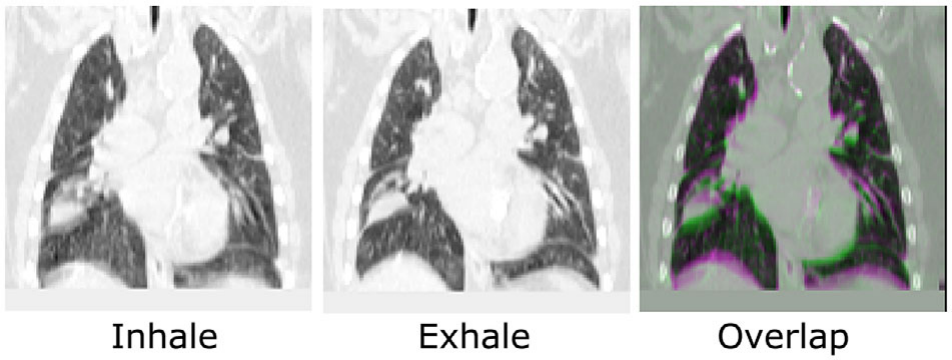
Illustration of the outlier case where the proposed algorithm achieved the lowest score. The inhale and exhale scans are shown (after cropping, padding, and resizing) in the first two images. The last image shows their overlap with the exhale image shown in green and the inhale in magenta. The CT scans have been padded at the bottom due to part of the lungs missing from the field of view. The combination of 4DCT artifacts together with the very limited motion between inhale and exhale resulted in all generated CTVI's showing almost no correlation with the reference SPECT scan. 4DCT, four‐dimensional CT; CT, computed tomography; CTVI's, CT‐ventilation imaging; SPECT, single photon emission computed tomography.

**FIGURE 4:**
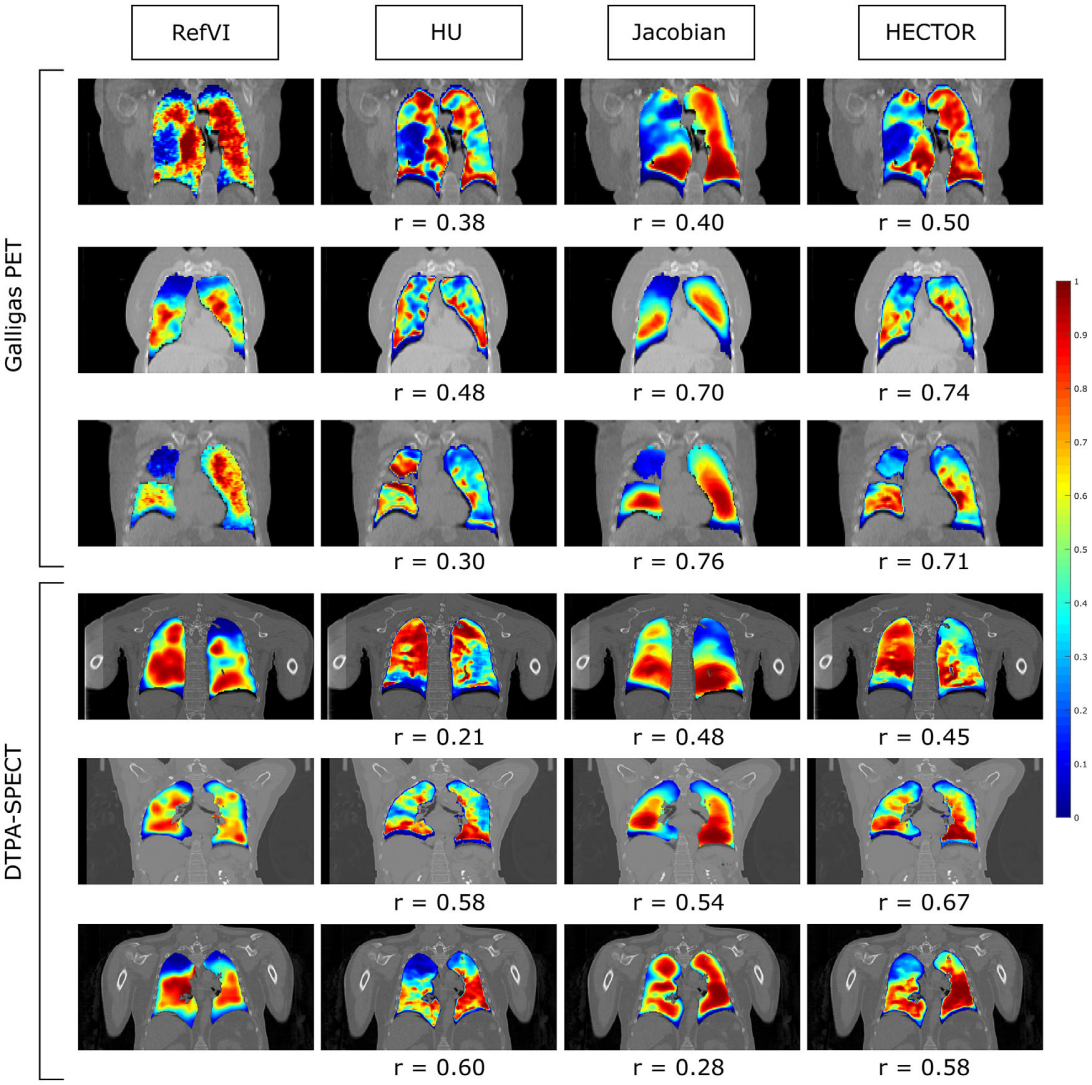
Example cases from the VAMPIRE dataset. Coronal slices of each method are shown together with the corresponding Spearman coefficient. Each row represents a different case. The first three cases are part of the Galligas PET sub‐study and correspond to cases 2, 13, and 22. The last three rows correspond to cases 2, 10, and 14 from the DTPA‐SPECT study. For illustration consistency, all ventilation maps were converted to ordering maps (see discussion after Equation 5) and were linearly rescaled in the interval [0,1]. The hybrid method depicts functionally deficit areas in the lung very similar to the reference ventilation maps. PET, pulmonary function test.

The results of the experiments are shown in Figure 2 while a statistical summary of the scores obtained on the test cases can be found in Table 1. Paired t‐tests were conducted to compare the Spearman coefficients of HECTOR and HU/Jacobian in the two test datasets. For the PET dataset, the results showed a statistically improved performance from HECTOR (P<.001 against both HU and Jacobian). For the DTPA‐SPECT test dataset, a significant difference was found between HECTOR and HU (P=0.002) but not between HECTOR and the Jacobian method (P=0.14). What is more, as can be seen in Table 1, the proposed method achieved (on average) a 17% higher Spearman coefficient on the PET sub‐study compared to the top performing VAMPIRE challenge entry, the biomechanicall model‐based DIR (BM‐DIR). Furthermore, we observed that all Spearman coefficients obtained using our hybrid method were higher than 0.41, with the exception of one outlier in the test data of the DTPA‐SPECT sub‐study. For this case, the generated CTVI's using all three methods (HU, Jacobian and HECTOR), presented almost no correlation with the reference SPECT scan. After retrospectively examining this case, it was found that the inhale/exhale CT scans exhibited very little motion and that the lower part of the lungs in the inhale scan was partially truncated. Those factors together with the presence of 4DCT artifacts resulted in the registration algorithm mislabeling the inferior part of the lungs as non‐functional. More details on this case can be found in Figure 3. To illustrate the qualitative differences of the various methods, selected examples displaying regional functional deficits are shown in Figure 4.

**TABLE 1:** Mean (standard deviation) of the Spearman coefficient, DSC‐high, and DSC‐low scores for the test cases of the VAMPIRE dataset.

Galligas PET				
	HECTOR	HU	Jacobian	BM‐DIR
**Spearman**	0.62 (0.10)	0.52 (0.13)	0.50 (0.14)	0.53 (0.10)
**DSC‐high**	0.55 (0.09)	0.51 (0.09)	0.45 (0.10)	0.47 (0.07)
**DSC‐low**	0.59 (0.07)	0.53 (0.09)	0.54 (0.08)	0.53 (0.08)

DTPA‐SPECT				
	**HECTOR**	**HU**	**Jacobian**	**BM‐DIR**
**Spearman**	0.49 (0.15)	0.35 (0.17)	0.44 (0.17)	0.49 (0.16)
**DSC‐high**	0.48 (0.10)	0.38 (0.12)	0.44 (0.11)	0.45 (0.11)
**DSC‐low**	0.50 (0.08)	0.46 (0.08)	0.47 (0.10)	0.52 (0.07)

*Note*: The results are divided as in the original study in two groups, corresponding to the Galligas PET substudy (20 test cases) and the DTPA‐SPECT substudy (11 cases). The reported values of the top performing method (BM‐DIR) submitted in the original challenge are also shown for comparison.

Abbreviations: HU, Hounsfield units; PET, pulmonary function test.

### 3.2 TCIA dataset

**FIGURE 5:**
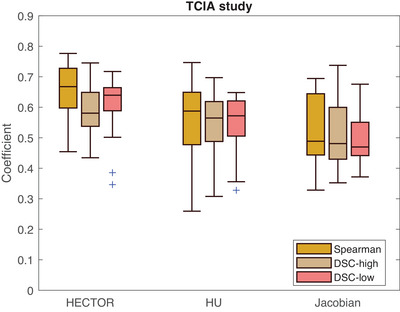
Results from the TCIA dataset. Boxplots of the three evaluated coefficients are shown for all the three algorithms used for a total of 18 cases. TCIA, The Cancer Imaging Archive.

**FIGURE 6:**
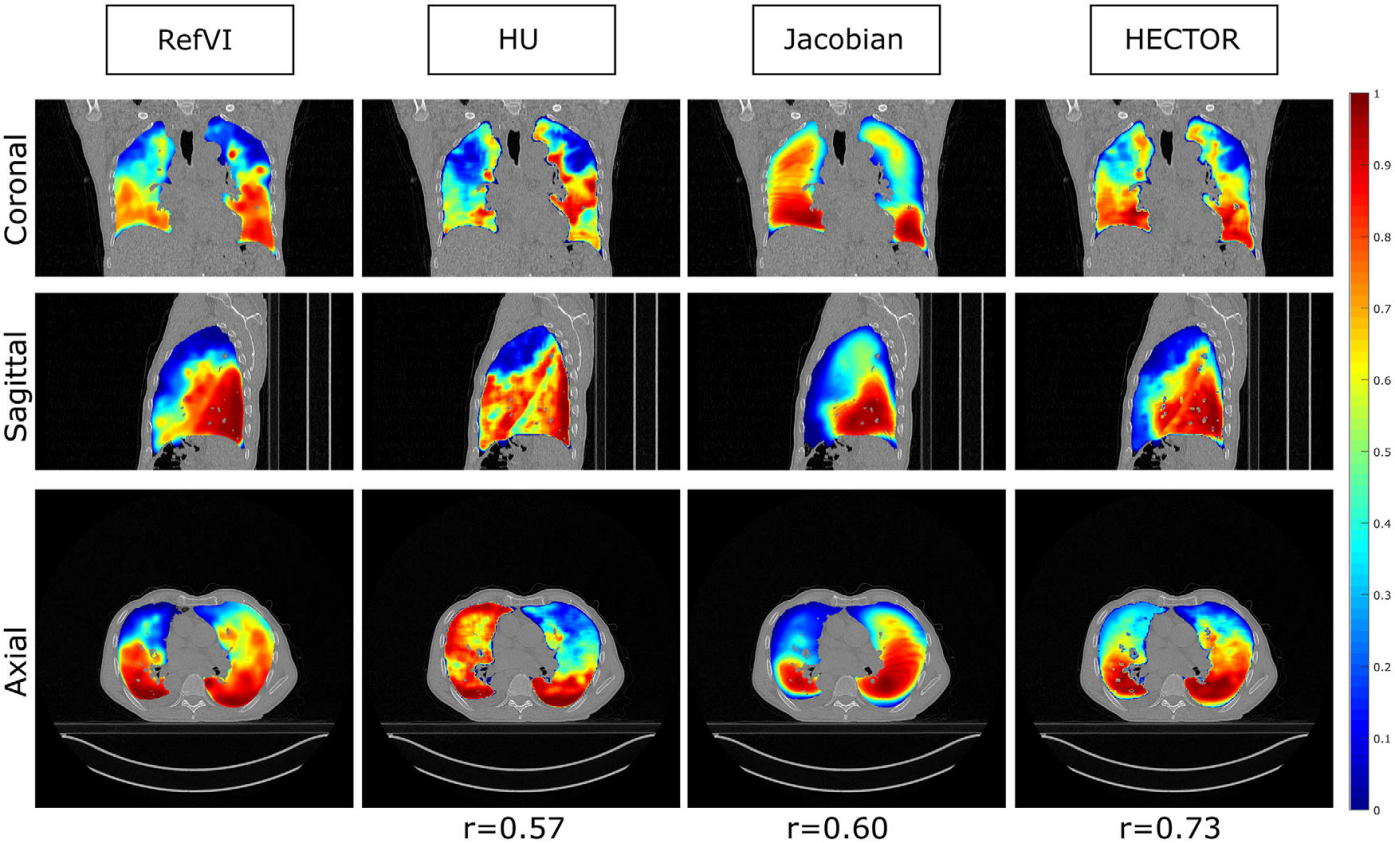
Example case (case 8) from the TCIA dataset. Coronal, sagittal and axial slices of each method are shown together with the corresponding Spearman coefficient. For illustration consistency, all ventilation maps were converted to ordering maps (see discussion after Equation 5) and were linearly rescaled in the interval [0,1]. We observe that HECTOR retains ventilation defects estimated by either the Jacobian or HU‐based methods. This results in improved correlation with the reference ventilation map. It can also be observed that both HU and Jacobian methods were prone to overestimating regional ventilation (false positives). This is the basis on which the use of the (smooth) minimum relies.

The results of the TCIA dataset are shown in Figure 5. We observe that the HU‐based metric performed better than the volume‐based one but worse than the combination of the two. In this dataset, there were no significant outliers for any of the three methods. The ranges of Spearman, DSC‐high and DSC‐low scores were similar to those of the VAMPIRE dataset. The mean (std) Spearman values were 0.55 (0.14), 0.52 (0.12), and 0.66 (0.09) and the lowest Spearman correlation values were 0.26, 0.33, and 0.45 for ${\text{CTVI}}_{\text{HU}}$, ${\text{CTVI}}_{\text{vol}}$, and ${\text{CTVI}}_{\text{HECTOR}}$, respectively. The results of paired t‐tests on the Spearman coefficients reveal a statistically significant difference between HECTOR and HU (P<.001) as well as between HECTOR and the Jacobian method (P<.001). An example case can be found in Figure 6 where coronal, axial, and sagittal slices of the three methods are shown together with the reference ventilation data.

### 3.3 Cumulative results

**FIGURE 7:**
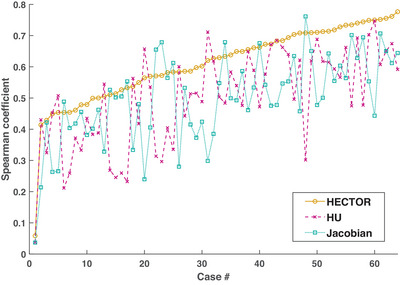
Cumulative Spearman correlation coefficient measures from both studies (64 cases in total) and for all the three ventilation methods. The cases have been sorted in order of ascending coefficients with respect to the HECTOR scores (yellow line). In 46 out of 64 cases (72%) the hybrid method scores the highest of the three, whereas in the remaining 18 cases (28%) the hybrid method is second. In none of the 64 reported cases does the proposed method score lower than both the Jacobian and HU methods. HECTOR, hybrid estimation of computed tomography obtained respiratory function; HU, Hounsfield units.

In order to provide some further insight in relation to the comparative scores of HECTOR and its individual components, Figure 7 shows the collective Spearman coefficients for all 64 cases tested and for all three methods. HECTOR scores the highest of the three methods in 46 cases (72%) while the Jacobian and HU achieved the highest score in 9 cases (14%) each. Furthermore, there was no case for which HECTOR scored worse than both its components.

## 4 DISCUSSION

We have presented a CT ventilation method that relies on the hypothesis of using complementary information provided by regional volumetric changes and static air‐tissue density measures. For this purpose, we have employed the GDC registration algorithm specifically tailored for accurately estimating local expansions/contractions inside the lungs by using a suitable regularization model inspired from physical principles. Furthermore, we have introduced a voxel ordering rescaling in order to transform arbitrary ventilation metrics into comparable data distributions. Finally, we proposed computing the voxel‐wise smooth minimum of the two given ventilation metrics (after the appropriate rescaling) to derive the new hybrid metric which efficiently combines information from both its constituents.

As can be seen in Table 1, our proposed method performs favorably compared to the top‐performing method^41^ in the original VAMPIRE challenge. The later method relies on a finite‐element model that uses vessel segmentations, organ contours, and elastic parameters of the various tissue types to generate biomechanically consistent deformations. Subsequently, it computes the stress map as a ventilation metric. This has proven to be a very robust approach as evidenced by the distinctively superior performance of the corresponding entry of the VAMPIRE challenge. We have demonstrated that using the proposed hybrid method, we are able to generate CT ventilation maps of similar and higher fidelity. Furthermore, the scores obtained on the VAMPIRE and TCIA datasets fall in a similar range, indicating a consistent behavior across different types of scans and relative to different ground truth data. What is more, the performance of the volume‐based metric ${\text{CTVI}}_{\text{vol}}$ was also found to be significantly better than previous studies have reported for similar techniques relying on the Jacobian determinant. Volume‐based methods have shown poor correlation with reference scans in emphysematous regions^42^ and have scored worse in the VAMPIRE challenge compared to HU‐metrics. Jacobian‐based CTVI methods have been known to be highly dependent on the registration algorithm used and this has been considered a crucial factor limiting their widespread implementation. The performance of DIR‐based CTVI methods depends on the accuracy of the registration method used. For example, if the registration accuracy is severely compromised (e.g., due to imaging artifacts), this could result in significant errors in the computed CTVI. This relationship has been rigorously investigated in the context of the VAMPIRE challenge.^28^ It was reported that the correlation between spatial accuracy (measured in terms of the target registration error) and the Spearman coefficient was weak overall but significant when considering certain algorithms and subject groups. In order to relax the dependence on the registration estimate, a previous study^43^ has demonstrated a highly reproducible method for acquiring volume‐based ventilation estimates. A key novelty was avoiding the use of finite difference derivatives. Our proposed workflow also requires no postprocessing differentiation of the deformation field since the GDC registration algorithm estimates directly the divergence that is used in the computation of Equation (3). We believe this to be one of the major factors contributing to the robustness of ${\text{CTVI}}_{\text{vol}}$ together with the physically‐inspired regularization model in Equation (1).

Different ventilation metrics often provide distinct information regarding regional pulmonary function and previous studies have also attempted to utilize a combination of more than one ventilation estimates. Some methods have introduced a tissue density scaling factor which models radioaerosol deposition^21^ and can be applied to correct both HU‐ and volume‐based metrics. In a similar fashion, some metrics rely on both intensity and volume information to derive ventilation maps, such as the *Specific air volume change by corrected Jacobian (SACJ)*.^44^ In this study, the combined SACJ metric was found to perform better than the individual intensity and volume components. Although in this case the coupling of the intensity‐based specific air volume change with the Jacobian determinant is dictated by physical arguments, in general there is no straightforward procedure for combining two given ventilation maps. In the present work, we have developed a framework for doing so, given two (or possibly more) arbitrary ventilation estimates. To do so, we introduced the crucial step of voxel ordering which renders arbitrary data distributions comparable to each other. Our hybrid ventilation metric was proposed on the basis of the observation that the smooth minimum amplifies the presence of defective regions whose identification is crucial for various clinical applications. Furthermore, we have experimentally observed that both ventilation metrics are more prone to overestimating ventilation (false‐positives) than underestimating it (false‐negatives). For example, in Table 1, the DSC‐low scores were higher than DSC‐high scores for all four methods reported. Therefore, from a modeling point of view, applying the voxel‐wise smooth minimum function in Equation (6) quantifies our confidence in lower ventilation estimates. An important finding of our work is that using the combined hybrid method results in significantly more robust ventilation estimation than using the individual Jacobian or HU metrics as summarized in Figure 7. From this figure, it can be inferred that the hybrid metric scores either higher than both of its two components or close to the highest of the two. Another conclusion drawn from the same figure is that, excluding the outlier case with poor scores due to inadequate motion in the anatomical data, all other Spearman correlation scores were higher than 0.41. For the purpose of functional avoidance radiotherapy, it has been reported^45^ that Spearman correlations above 0.4 may be sufficient for producing concordant CTVI‐based and SPECT‐based functional treatment plans. In light of this study and the rest of our findings, we believe that the proposed method can potentially be utilized as a surrogate to nuclear medicine methods for lung functional avoidance. At the same time, we strongly argue that further studies on clinical data are needed in order to advance our understanding on the conditions under which a CTVI is sufficiently accurate to be used in clinical applications. This line of research could also lead to the development of suitable quality criteria for the generated ventilation maps, which are currently missing.

It is important to point out some limitations of the present study. To begin with, we observed that the worst scores were obtained for 4DCT datasets of poor quality due to respiratory binning artifacts, as a result of the inferior part of the lungs being truncated or due to very small scale motion present in the CT scans as in the worst case shown in Figure 3. Those issues can be addressed by using well‐tailored acquisition methods and adjusting the field of view to ensure that the entire pulmonary volume is visible. The importance of using appropriate images has been previously highlighted.^33^ Moreover, our use of voxel‐ordered maps to combine different physical quantities (air‐tissue density and volume changes) deprives our functional maps of physical units. After ordering, the functional state of a voxel is only interpretable relative to the rest of the lungs. This is sufficient for our intended purpose in FLART, where the goal is usually the identification of the most and least functional regions. However, for other applications, such as population studies, where functional data from different individuals are compared, more refined strategies need to be employed. This is a common shortcoming of synthetically generated data and we believe that further research is needed in this direction. With respect to the validation process, we expect possible small misalignments of the RefVI's with the exhale anatomy on which the CTVI is computed. This was a potential source of error in both datasets used in this work. For the VAMPIRE dataset, the RefVI's were obtained during free breathing and were therefore aligned with the average anatomy. For the TCIA dataset, we aligned the ventilation scans with the anatomical images using a combination of mutual information (MI)—based affine registration and manual corrections. What is more, our validation has been carried out using nuclear medicine methods as ground truth. The later are known to be prone to clumping artifacts (SPECT) and high noise levels (PET). An alternative validation has been investigated,^46^ where imaging and pathology findings were compared for the assessment of post‐RT respiratory function changes. Such studies, offer crucial insight into the degree of accuracy of the developed computational methods but are inherently difficult to carry out.

## 5 CONCLUSION

We have presented HECTOR, a novel CT ventilation imaging method that employs the (smooth) minimum of two ventilation metrics, a volume‐based and a HU‐based, to generate functional maps that highlight defective regions present in either of the inputs. The volume‐based component relies on the GDC image registration algorithm that was found to improve the accuracy of the estimated volume changes. Our results indicate that the hybrid combination is advantageous over its components in terms of all the evaluation metrics tested. This finding corroborates the hypothesis that, due to the complexity of ventilation dynamics, different types of data can efficiently be combined in order to accurately capture the physiological information that is relevant for respiratory function.

## CONFLICT OF INTEREST STATEMENT

The authors have no relevant conflicts of interest to disclose.
